# Genetic variation in compensatory feeding for dietary dilution in a generalist caterpillar

**DOI:** 10.1038/s41598-017-07822-4

**Published:** 2017-08-07

**Authors:** Kwang Pum Lee

**Affiliations:** Department of Agricultural Biotechnology, Seoul National University, Seoul, 08826 Republic of Korea

## Abstract

Increasing the rate of food consumption is a common adaptive strategy that allows herbivores to compensate for declines in nutrient concentrations in plant tissues. Herbivores that are better able to compensate for dietary dilution may have selective advantages under nutritionally poor conditions. In order for compensatory feeding to respond to selection, there must be standing heritable variation for this trait. However, empirical data substantiating the adaptive significance and genetic variability of compensatory feeding are rare. By employing a full-sib, split-brood design, this study presents quantitative genetic analyses on the nutrient consumption rates of the generalist caterpillar, *Spodoptera exigua*, raised on semi-synthetic diets differing in nutrient concentrations. When encountering a diluted diet, caterpillars exhibited a compensatory increase in food consumption rate, but the extent of this increase was not sufficient to fully compensate for dietary dilution. A significant gene-environment interaction for consumption rate indicated that the capacity of caterpillars to compensate for dietary dilution varied across genotypes. The broad-sense heritability of compensatory feeding was 0.51. Caterpillar genotypes with a higher compensatory capacity suffered lower performance losses on the diluted diet than did those with a lower capacity. This study has implications for understanding how herbivores can evolutionarily respond to nutritional challenges.

## Introduction

Under natural conditions, herbivores are constantly confronted with suboptimal food conditions due to the deficiency of essential nutrients in plant tissues^1, 2^. The chronic ingestion of low-quality food reduces nearly all aspects of consumer performance and thus poses a major challenge for herbivores especially when they are restricted from accessing alternative food sources^3–6^. Many herbivores have developed a highly conserved compensatory mechanism which enables them to counteract the low nutrient content of the food by increasing the rate of food consumption^7–9^. Compensatory feeding is thus a trait of great ecological importance and may have been favoured by natural selection in herbivores. One important requirement for the evolution of compensatory feeding to occur is the presence of its standing genetic variation. However, no study to date has explicitly assessed the genetic variation in compensatory feeding of herbivores in response to nutritionally diluted foods.

This study presents a quantitative genetic analysis on the consumption rate of a generalist leaf-chewing caterpillar feeding on semi-synthetic diets with two different nutrient concentrations. The particular species of caterpillar used in this study was *Spodoptera exigua* (Lepidoptera: Noctuidae), a major agricultural pest found in temperate and subtropical regions around the world^10^. The nutrient concentration of the experimental diets was manipulated by replacing 0% or 50% nutrients with cellulose, a major plant structural compound that is indigestible to most insects including caterpillars^11^. Together with other structural compounds such as hemicellulose and lignin, cellulose comprises a large fraction of plant tissues (for cellulose alone, ca. 16~47% of plant tissues by dry mass) and is known to have profound effects on herbivores by diluting the nutrient concentration of plant tissues^12^.

There are two specific questions to be addressed in this study. First, is there any heritable variation in compensatory feeding for dietary dilution? This question was answered by determining a significant gene-environment interaction (GEI, henceforth) for consumption rate and then by calculating the broad-sense heritability for the plasticity of consumption rate across dietary environments^13^. Second, is compensatory feeding really adaptive? The adaptive significance of compensatory feeding lies in the fact that it enables consumers to alleviate the fitness-reducing effects of inferior food quality^7^, but empirical studies corroborating the beneficial consequences of compensatory feeding are surprisingly rare^14^. To gain further evolutionary insights into the adaptive significance of compensatory feeding, it is necessary to demonstrate whether the capacity of caterpillars to compensate for dietary dilution is genetically correlated with their capacity to reduce the negative consequences of ingesting nutritionally diluted diets.

## Methods

### Study organism and rearing

A large, outbred laboratory population of *S. exigua* was established from several hundred caterpillars collected at various field sites near Suwon (37° 17′ N, 127° 00′ E), Republic of Korea, in 2009. Field-collected insects were brought to Seoul National University where they had been maintained on a standard, semi-synthetic rearing diet for 17 generations prior to the experiment. In order to maintain high levels of genetic variation of the population, more than 300 full-sib families were generated at each generation by randomly pairing unrelated male and female virgin moths^15^. Each pair was allowed to mate and lay eggs in a transparent plastic arena (120 mm diameter, 80 mm height) containing 10% sucrose solution and oviposition substrate (paper towel). Eggs laid on the substrate were collected and incubated at 25 °C. Newly hatched caterpillars from these eggs were transferred to Petri dishes (90 mm diameter) in which they were reared in groups of 40–50 insects up to the end of their fourth larval instar. During the final (fifth) instar, however, caterpillars were individually confined in the wells of 24-well plates until pupation. Food was supplied ad libitum to caterpillars throughout the larval rearing period. Pupae were sexed and placed individually in a plastic cup (50 mm diameter, 40 mm height) for emergence. All culture rearing was conducted at 25 °C and 12:12 L:D photoperiod.

### Experimental diets

Following Goh *et al*.^16^, two semi-synthetic diets differing in the total concentrations of dry nutritive ingredients (e.g., wheat germ, kidney bean powder, brewer’s yeast, and Wesson’s salt; see Table S1) were prepared, one containing the standard concentrations of these nutritive ingredients (undiluted diet) and the other containing only the half of the standard level (50% diluted diet). In the latter diet, the remaining half of the dry mass was filled with cellulose (α-cellulose, Sigma C8002), a non-nutritive bulking agent that is indigestible to caterpillars^11^. Nutritive ingredients (e.g., wheat germ, kidney bean powder, brewer’s yeast, and Wesson’s salt) were the sources of dietary nutrients (e.g., protein, carbohydrate, lipids, and micronutrients) and so were collectively referred to as ‘nutrients’ although they may also include naturally occurring indigestible material^17^. Experimental diets were prepared by homogenously suspending all dry constituents (253.3 g see Table S1) in 600 mL of a 1.5% agar solution followed by autoclaving at 110 °C for 20 min. When the agar media had cooled to <50 °C, the constant quantities of vitamin (ascorbic acid) and preservatives (sorbic acid, formalin, and methyl-p-hydroxybenzoate; see Table S1) were added and the mixture was stirred vigorously. Solid diets were then dispensed into plastic containers, stabilized at room temperature for 6 h, and stored at 4 °C before use.

### Experimental design

The relative contribution of genetic and environmental (diet) factors to phenotypic variation in consumption rates in *S. exigua* caterpillars was investigated by employing a full-sib, split-brood design where caterpillars from each of the 48 full-sib families were divided into two diet groups. For each full-sib family, caterpillars were reared on a standard rearing diet at a density of 20–30 insects in a Petri dish (90 mm diameter) until reaching the end of the fourth instar. On moulting to the final (fifth) instar, caterpillars from each family were randomly split over two diet groups (0% and 50% diluted diets), with an average of 4.53 insects for each family × diet combination. Each caterpillar was then individually confined to a Petri dish (60 mm diameter) and received a block of either undiluted or 50% diluted diet. The fresh mass of each block was ca. 1.2 g, an amount that exceeds the daily consumption of this caterpillar. Diet blocks were replaced daily and uneaten remains were removed, dried to constant mass at 50 °C, and then weighed to the nearest 0.1 mg using an electronic microbalance (Ohaus Co., Parsippany, NJ, USA). This procedure was repeated until all caterpillars ceased to feed upon entering their prepupal stage. For each diet group, 20 control diet blocks were weighed, dried, and reweighed to construct a regression equation from which the initial dry mass of diet blocks was back-calculated. Daily food consumption was determined as the difference between the initial and final dry mass of each diet block. Nutrient consumption was then calculated as the product of food consumption and nutrient concentration. The total amount of nutrients consumed over the first two days of the final instar (days 0–2) was taken as a measure of nutrient consumption rate. Consistent with earlier studies using caterpillars of related species (e.g., *Spodoptera litura* ^18^), the pattern of consumption was statistically indistinguishable between males and females during this early stage of the final instar (sex effect in ANOVAs conducted separately for all full-sib families: *p* > 0.78). Accordingly, the data for nutrient consumption rate obtained from the two sexes were pooled to increase statistical power. The duration of the final instar was recorded to the nearest day. Pupae were sexed, frozen to death, dried to constant mass, and weighed to the nearest 0.1 mg. The dry mass of caterpillars at the start of the final instar was estimated by a regression equation constructed from 30 control caterpillars that were randomly collected across full-sib families (*R* ^2^ = 0.97). Growth rate was calculated as the amount of body mass increased during the final instar (dry mass) divided by the instar duration (days). Throughout the experiment, all insects were maintained in an environmental chamber set at a constant temperature of 25 °C and 12:12 L:D photoperiod.

### Data analysis

All statistical analyses were conducted with SAS version 9.12 (SAS Institute, Cary, NC, USA). Two-way factorial ANOVA was performed to test the effect of diet, full-sib family, and their interaction on nutrient consumption rate by using PROC GLM, with full-sib family being designated as random effect. To determine the diet-specific heritability of nutrient consumption rate, one-way ANOVA was performed separately for each diet group. If a significant effect due to full-sib family (*p* < 0.05) was detected in this analysis, variance component terms were subsequently estimated using the restricted maximum likelihood (REML) method implemented in PROC VARCOMP. Broad-sense heritability (*H* ^2^) was calculated using the following standard formula for full-sib design, ${H}^{2}=\frac{2\times {V}_{AF}}{{V}_{P}}=\frac{2\times {V}_{AF}}{{V}_{AF}+{V}_{WF}}$where *V*_*P*_ is the total phenotypic variance, *V*_*AF*_ the among-family variance, and *V*_*WF*_ the within-family or error variance^19^. Broad-sense heritability obtained from this full-sib design should be treated as an upper limit to the actual value because it may also contain non-additive genetic (e.g., dominance and epistasis), maternal, and common environmental effects^19^. Standard errors for this heritability were estimated using the formula provided in Roff^19^. Any significant cross-environment difference in the estimated heritabilities was tested using the two-sample *z*-test^20^.

In cases where family × diet interaction (GEI) was significant, reaction norms were plotted to visualize how genotypes (i.e., full-sib families) varied in their responses to dietary dilution^19, 21, 22^. Spearman rank correlation on family mean values across diets was performed to determine whether GEI had a significant crossover^21^. Following the logic of Windig^23^, the heritability of trait plasticity was calculated as the proportion of the total phenotypic variance in slopes of linear reaction norms attributable to the additive genetic variance. A bootstrap procedure was applied to estimate this broad-sense heritability for the slopes by taking the following steps^23^. First, for each family, four individuals were randomly drawn from one diet group with replacement and each was then arbitrarily paired with one of four individuals that were also randomly sampled from the other diet group. This random pairing generated a total of 192 slopes from 48 full-sib families (four per family). Second, from this randomly sampled data set, variance component terms were obtained using the REML method and broad-sense heritability (*H* ^2^) for the slope was calculated using the standard formula for full-sib design as described above. Third, the whole process was repeated 1,000 times, with the mean of 1,000 replications being taken as the estimate of heritability (*H* ^2^).

Genetic correlation between the measurements of the same trait (nutrient consumption rate) in two dietary environments (i.e., cross-environment genetic correlation) was calculated as *r*_g_ = Cov[*u*,*d*]/(*V*_*u*_ × *V*_*d*_)^0.5^ where *V*_*u*_ and *V*_*d*_ are the genetic variance of the same trait expressed in undiluted and 50% diluted diet, respectively, and Cov[*u*,*d*] is the genetic covariance between the same traits expressed in two diet groups^24^. Variances, covariance, and their standard errors used for calculating genetic correlations (*r*_*g*_) across dietary environments were obtained using the REML method in PROC MIXED^25^. Likelihood-ratio tests were used to determine whether these estimated genetic correlations were significantly different from 0 (two-tailed) or 1 (one-tailed). In this test, twice the difference in log-likelihoods between a full model and a model in which *r*_g_ was constrained to 0 or 1 was used as the test statistic that follows a χ^2^ probability distribution with the degree of freedom of 1.

The degree to which caterpillars compensated for dietary dilution was represented by family-mean compensation index (CI), which was calculated as CI = ln(*C*_*d*_/*C*_*u*_) where *C*_*u*_ and *C*_*d*_ are the family mean amount of nutrient eaten over days 0–2 by caterpillars on undiluted and 50% diluted diet, respectively. A value of 0 for this index indicates that caterpillars fully compensate for dietary dilution while a negative value indicates that the dietary dilution is not fully compensated by caterpillars. The more negative the value for CI, the lesser the degree of compensation for dietary dilution. Similarly, the degree to which the performance (pupal mass, growth rate, or instar duration) of caterpillars was negatively affected by dietary dilution was represented by family-mean performance-change index (PI), which was calculated as PI = ln(*P*_*d*_/*P*_*u*_) where *P*_*u*_ and *P*_*d*_ are the family mean performance of caterpillars on undiluted and 50% diluted diet, respectively. A value of 0 for this index means that there is no effect of dietary dilution on performance. To test whether the capacity of caterpillars to compensate for dietary dilution was associated with their capacity to buffer the negative impact of dietary dilution on performance, family-mean correlations between CI and PI were conducted using the Pearson’s product moment correlation, with the coefficient of correlation and its standard error being estimated using the delete-one jackknife^26^.

## Results

### Genetic variation in compensatory feeding

**Figure 1:**
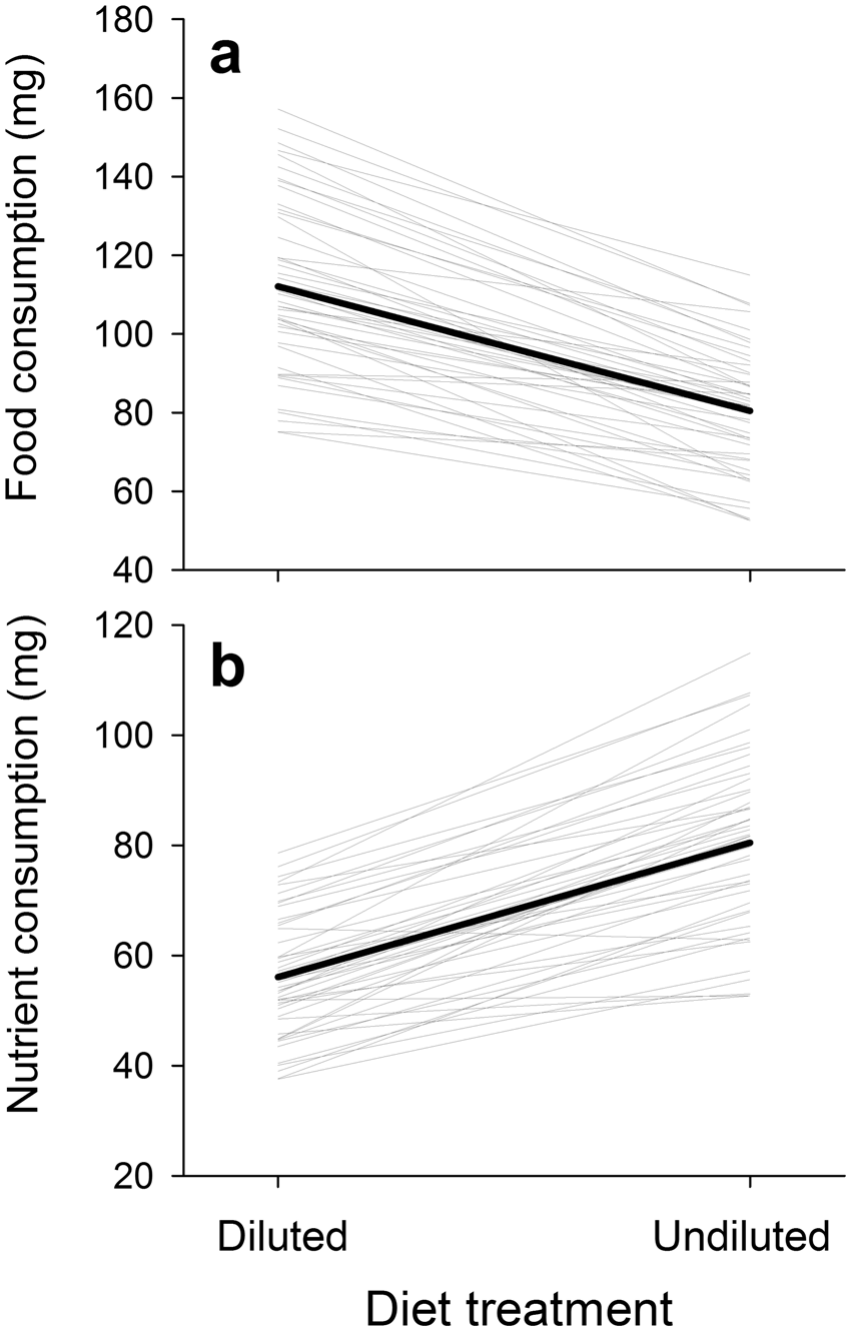
Gene-environment interaction for the amount of (**a**) food and (**b**) nutrient consumed by *Spodoptera exigua* over the first two days of the final larval instar in two dietary environments (undiluted and 50% diluted diet). For each trait, reaction norms (thin grey lines) are plotted by connecting the trait means of 48 full-sib families in two environments. In each panel, the overall means of each measured trait in two environments are connected by thick black lines to demonstrate the overall diet effect.

Caterpillars that were restricted to a 50% diluted diet consumed ca. 39.9% more food over days 0–2 than did those restricted to to an undiluted diet (Fig. 1a). However, the amount of cellulose-free nutrients eaten over the same period was ca. 30.1% lower on a 50% diluted diet than on an undiluted diet (Fig. 1b; ANOVA: *F*_1,340_ = 399.24, *p* < 0.001), suggesting that compensation for dietary dilution was only partially complete. The genetic variability of nutrient consumption rate in the population was confirmed by a significant effect of full-sib family on this trait (*F*_47,340_ = 8.64, *p* < 0.001). As summarized in Table 1, the broad-sense heritability (*H* ^2^ ± SE) of nutrient consumption rate was estimated to be 0.929 ± 0.146 and 0.975 ± 0.143 on undiluted and 50% diluted diet, respectively. There was no significant difference in heritability for this trait across diets (two-sample *z*-test: *z* = 0.228, *p* = 0.820). As indicated by a significant full-sib family × diet interaction (GEI) for nutrient consumption rate (*F*_47,340_ = 1.5, *p* = 0.024), the slopes of reaction norm for this trait varied significantly among full-sib families (Fig. 1b). The broad-sense heritability of this trait plasticity (i.e., the slope of reaction norm; *H* ^2^ ± SE) was 0.509 ± 0.129 (see Table 1). Despite the significant GEI, however, the plasticity was not strong enough to alter the overall rank order of family-mean consumption rate across diets (Spearman rank correlation: *ρ* = 0.750, *P* < 0.001). Cross-environment genetic correlation (*r*_*g*_ ± SE) for nutrient consumption rate was 0.958 ± 0.070, which was significantly different from 0 (likelihood-ratio test, two-tailed: χ^2^ = 38.2, df = 1, *p* < 0.001) but not from 1 (one-tailed: χ^2^ = 0.4, df = 1, *p* = 0.264).

**Table 1:** The summary of among-family variance component (*V*_*AF*_), within-family variance component (*V*_*WF*_), total phenotypic variance component (*V*_*P*_), and broad-sense heritability (*H* ^2^ ± SE) estimated for nutrient consumption in two dietary environments (undiluted and 50% diluted diet) and its trait plasticity across environments in *Spodoptera exigua*.

	*V*_*AF*_	*V*_*WF*_	*V*_*P*_	*H* ^2^ ± SE	*p*
Undiluted diet	188.407	217.389	405.796	0.929 ± 0.146	<0.001
Diluted diet	95.901	100.784	196.685	0.975 ± 0.143	<0.001
Trait plasticity	89.673	261.049	350.722	0.509 ± 0.129	<0.001

Nutrient consumption was measured as the amount of nutrient consumed over the first two days of the final instar. The *p*-value of heritability for nutrient consumption on each diet was derived from the effect of family in one-way ANOVA whereas that for its trait plasticity was computed by running permutation test for one-way ANOVA with 10,000 permutations.

### The adaptive significance of compensatory feeding

**Figure 2:**
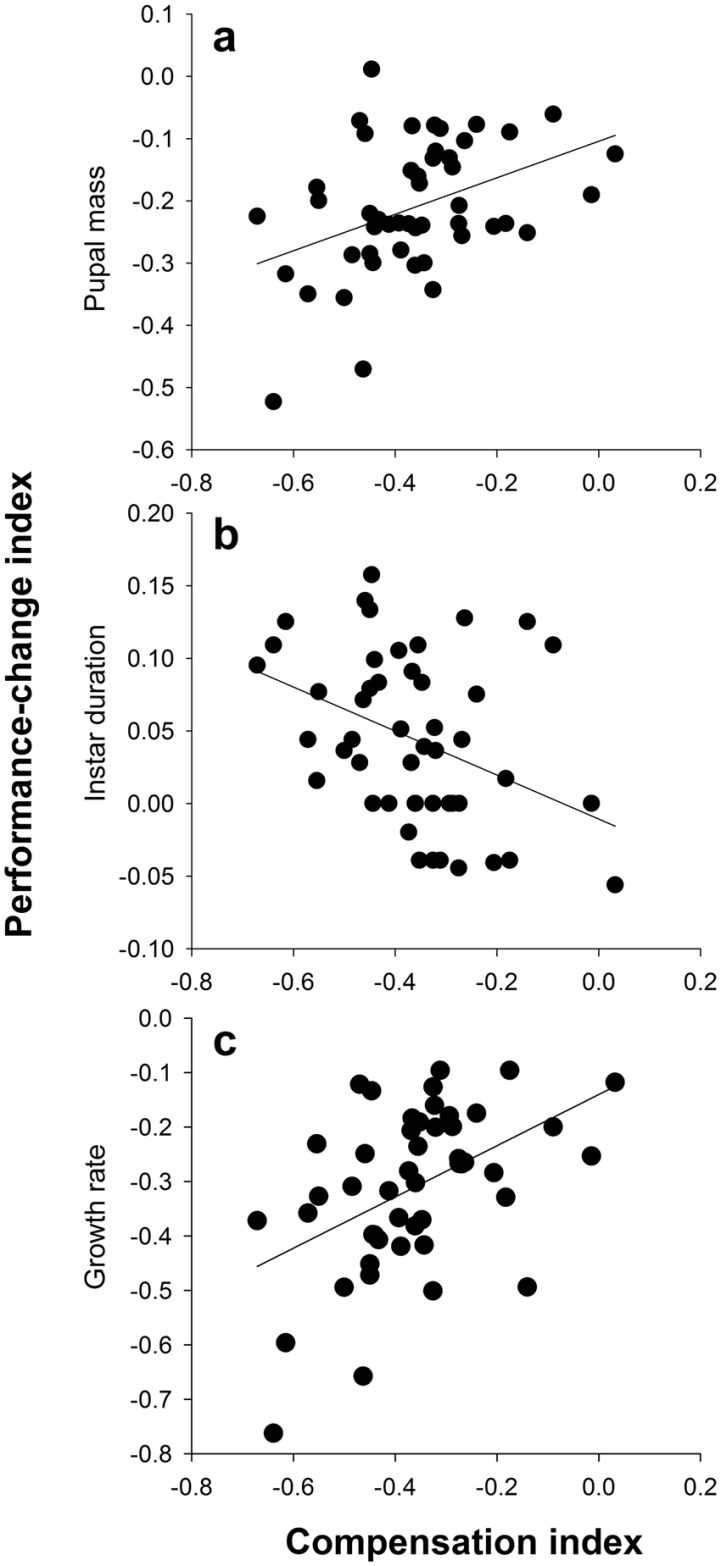
The relationship between the degree of compensatory feeding for dietary dilution (compensation index, CI) and the degree of changes in performance due to dietary dilution (performance-change index, PI) for (**a**) body mass, (**b**) instar duration, and (**c**) growth rate over 48 full-sib families in *Spodoptera exigua*. Each symbol represents the family-mean value for PI and CI. Least-squares linear regressions were fitted to describe the relationship between CI and each aspect of PI (**a**, body mass: *y* = −0.104 + 0.293 *x*, *R* ^2^ = 0.167, *p* = 0.004; **b**, instar duration: *y* = −0.011–0.152 *x*, *R* ^2^ = 0.149, *p* = 0.007; **c**, growth rate: *y* = −0.140 + 0.470 *x*, *R* ^2^ = 0.223, *p* < 0.001).

Caterpillars raised on a 50% diluted diet had 3.7% longer instar duration (undiluted vs. 50% diluted diet, mean ± SE: 5.17 ± 0.060 days vs. 5.36 ± 0.069 days; paired *t*-test: *t*_47_ = 3.69, *p* < 0.001), 18.8% smaller body mass at pupation (27.72 ± 0.299 mg vs.22.51 ± 0.315 mg; *t*_47_ = 14.10, *P* < 0.001), and 26.2% lower growth rates (4.24 ± 0.072 mg/day vs.3.13 ± 0.068 mg/day; *t*_47_ = 15.87, *p* < 0.001) compared with those raised on an undiluted diet. The extent to which these three components of larval performance (body mass at pupation, instar duration, and growth rate) were reduced by dietary dilution varied considerably among full-sib families (Fig. 2). The family-mean CI (i.e., the index representing the capacity of caterpillars to ingestively compensate for dietary dilution) was positively correlated with the family-mean PI (i.e., the index representing the capacity of caterpillars to buffer the negative effects of dietary dilution on performance) calculated for body mass (family-mean correlation: *ρ* = 0.405 ± 0.124, *p* = 0.004; Fig. 2a) and for growth rate (*ρ* = 0.486 ± 0.123, *p* < 0.001; Fig. 2c), but was negatively correlated with that for instar duration (*ρ* = −0.404 ± 0.139, *p* = 0.007; Fig. 2b).

## Discussion

### Genetic variation in compensatory feeding

Compensatory increase in food consumption is the most common adaptive response exhibited by herbivores upon encountering plant tissues with low nutrient concentrations^7, 8^. Consistent with this general pattern, the present study showed that caterpillars significantly increased their consumption rate in response to dietary dilution, but the extent of this increase in food consumption was not sufficient enough to fully compensate for the 50% dilution of the food. The fact that the caterpillars have limited ability to compensate for dietary dilution is largely consistent with previous studies^17, 27–30^ and indicates that there is an upper limit to the extent to which caterpillars can increase their consumption of nutritionally diluted food^7, 8^. Physiological constraints preventing the complete compensation may include the negative volumetric feedback caused by increased occupation of cellulose-diluted diet in the gut^8, 9^ and the reduced phagostimulatory power of the nutritionally diluted food^9, 31^.

As indicated by a significant effect of full-sib family on nutrient consumption rate, this study confirmed that there was a substantial amount of standing genetic variation for this trait in the population. Broad-sense heritability (*H* ^2^) was calculated to be 0.929–0.975 for nutrient consumption rate across two diets, raising the possibility that this trait will strongly respond to selection. However, these extremely high heritabilities estimated using full-sib breeding design should be interpreted with some caution because they might be inflated by the inclusion of non-additive genetic (i.e., dominance and epistasis) and maternal effects^19^.

There was a significant full-sib family × diet interaction for nutrient consumption rate, indicating that the slopes of reaction norm for this trait differed among full-sib families (see Fig. 1). This significant GEI can be taken as evidence for the significant genetic variation in the phenotypic plasticity for consumption rate (i.e., compensatory feeding). While previous studies have reported that the extent to which herbivores can compensate for diluted nutrient varies both inter- and intra-specifically^7, 8, 13, 32, 33^, to the best of my knowledge, this is the first time to demonstrate standing genetic variation in compensatory feeding. The presence of substantial among-family variation in the ability to compensate for dietary dilution indicates that the strength of the physiological constraints operating on compensatory feeding may also vary across genotypes.

According to quantitative genetic theories, traits tightly associated with fitness are predicted to possess lower heritabilities than those loosely linked to fitness^34^. Since compensatory feeding is an important fitness trait that enables herbivores to reduce fitness losses under nutritionally stressful conditions, one might expect a low heritability for this trait. Contrary to this expectation, however, the broad-sense heritability of compensatory feeding was estimated to be high (*H* ^*2*^ = 0.509). Such high levels of genetic variation in compensatory feeding could have been maintained within natural populations through a number of mechanisms, including mutation, heterozygous advantage, antagonistic pleiotropy, frequency-dependent selection, environmental heterogeneity, etc^19^. Despite its potential selective advantages, compensatory feeding may entail some ecological costs which can contribute to the maintenance of the genetic variation of this trait within natural populations. For example, compensatory increases in the duration and frequency of feeding on nutritionally diluted diet may lead caterpillars to suffer high risk of predation^35^. It is also possible that increased intake of nutritionally diluted plant tissues may intoxicate caterpillars by concomitantly increasing the ingestion of toxic allelochemicals or environmental toxicants present in plants to lethal doses^29, 36^.

Besides analysing the pattern of the reaction norms plotted across two environments, the genetic basis of phenotypic plasticity can be also investigated through taking the character state approach, which considers the same trait measured in discrete environments (diets in this case) as separate ‘character states’ that are genetically correlated^19, 22^. Accordingly, in the current study, nutrient consumption rates expressed in two dietary environments were treated as two character states and the genetic correlation between these character states was determined to predict whether or not these character states were genetically independent^24, 37^. It is generally argued that any cross-environment genetic correlation significantly deviating from 0 will constrain the independent evolution of character states expressed in each of the heterogeneous environments^22^. The present finding that the cross-environment genetic correlation estimated for nutrient consumption rate was statistically equal to +1 implies that the same gene or set of genes were involved in the expression of this trait in two environments in the same way and further suggests that nutrient consumption rates in two dietary environments will be completely prevented from reaching their respective local optima.

### The adaptive significance of compensatory feeding

In order to investigate the performance consequences of compensatory feeding across full-sib families, three parameters representative of larval performance were measured from 48 full-sib families raised on two diets: body mass at pupation, instar duration, and growth rate. There is ample empirical evidence that these performance parameters are strongly linked to fitness in larval Lepidoptera. For example, small body size or mass at maturity results in reduced fecundity in both males and females^38^ and extended larval period causes high larval mortality by increasing predation risk^39, 40^. As generally expected, the results of this study showed that caterpillars on nutritionally diluted diet had prolonged instar duration, reduced body mass, and slowed growth rates compared with those fed on an undiluted diet, suggesting that there were significant performance losses caused by the ingestion of nutritionally diluted diets^27–30^.

The capacity of caterpillars to mitigate the negative consequences of dietary dilution was found to be genetically variable. The results of the family-mean correlation conducted between CI and PI revealed that the extent to which dietary dilution reduced growth rate and body mass at maturity was lower for caterpillar families with a greater ingestive capacity to compensate for dietary dilution. Similarly, the extent to which the duration of the final instar was prolonged by dietary dilution tended to decrease as the degree of compensation for dietary dilution increased. Collectively, these results suggest that compensatory increase in food intake enables herbivores to buffer the negative effects of dietary dilution on performance and thus may confer selective advantages to herbivores under nutritionally stressful conditions. From an ecological perspective, higher capacity to compensate for dietary dilution may indicate less need for switching host plants, thus reducing exposure to predators^14, 41^.

### Conclusion and prospectus

In conclusion, this study substantiates the genetic variability and adaptive significance of compensatory feeding for dietary dilution and provides important empirical support to the notion that compensatory feeding can rapidly evolve and respond to natural selection. While compensatory increase in feeding rate was the sole focus of the present study, herbivores are also known to compensate for reduced nutrient intake through increasing post-ingestive nutrient assimilation efficiencies^7–9^. Therefore, future studies should be directed at determining the genetic variance and covariance of post-ingestive compensatory response of herbivores to varying nutrient concentrations in the food. One of the most challenging nutritional obstacles faced by herbivores under recent climate change is the reduction of specific nutrients, such as nitrogen, in the plant tissues^42, 43^. The findings of the present study thus have important implications for predicting how rapidly herbivores can adapt to changes in the nutritional environments driven by climate warming.

### Data availability statement

The datasets generated and analysed during the current study are available from the corresponding author on reasonable request.

## Electronic supplementary material

Supplementary Information
