# Supplementary material for: Genetic variation in compensatory feeding for dietary dilution in a generalist caterpillar
Source: Sci Rep. 2017 Aug 7;7:7461. doi: 10.1038/s41598-017-07822-4 (PMC5547126; doi:10.1038/s41598-017-07822-4)
Supplement: Supplementary file 1 — Supplementary Information [file 41598_2017_7822_MOESM1_ESM.pdf]

Genetic variation in compensatory feeding for dietary dilution in a generalist caterpillar

Kwang Pum Lee[1, *]

[1] Department of Agricultural Biotechnology, Seoul National University, Seoul 08826, Republic of Korea

[*] Correspondence: kwanglee@snu.ac.kr

Supplementary information.

Table S1. Composition of the two semi-synthetic diets (undiluted and 50% diluted diet) used in the experiment.

Ingredient	Undiluted diet	Diluted diet
Wheat germ (g)	100	50
Kidney bean powder (g)	100	50
Brewer's yeast	53.3	26.65
Wesson's salt (g)	6.7	3.35
Cellulose powder (g)	0	130
Ascorbic acid (g)	12	12
Sorbic acid (g)	2	2
Methyl-p-hydroxybenzoate (g)	4	4
Formalin (mL)	4	4
Agar (g)	9.3	9.3
Distilled water (mL)	600	600
